# Species-Specific Duplication of Surface Antigen Genes in *Paramecium*

**DOI:** 10.3390/microorganisms10122378

**Published:** 2022-11-30

**Authors:** Marcello Pirritano, Yulia Yakovleva, Alexey Potekhin, Martin Simon

**Affiliations:** 1 Molecular Cell Biology and Microbiology, School of Mathematics and Natural Sciences, University of Wuppertal, 42119 Wuppertal, Germany; 2 Laboratory of Cellular and Molecular Protistology, Zoological Institute of Russian Academy of Sciences, 190121 Saint Petersburg, Russia; 3 Research Department for Limnology, University of Innsbruck, 5310 Mondsee, Austria

**Keywords:** antigenic variation, *Paramecium*, surface antigen, multigene family

## Abstract

*Paramecium* is a free-living ciliate that undergoes antigenic variation and still the functions of these variable surface antigen coats in this non-pathogenic ciliate remain elusive. Only a few surface antigen genes have been described, mainly in the two model species *P. tetraurelia* strain 51 and *P. primaurelia* strain 156. Given the lack of suitable sequence data to allow for phylogenetics and deeper sequence comparisons, we screened the genomes of six different *Paramecium* species for serotype genes and isolated 548 candidates. Our approach identified the subfamilies of the isogenes of individual serotypes that were mostly represented by intrachromosomal gene duplicates. These showed different duplication levels, and chromosome synteny suggested rather young duplication events after the emergence of the *P. aurelia* species complex, indicating a rapid evolution of surface antigen genes. We were able to identify the different subfamilies of the surface antigen genes with internal tandem repeats, which showed consensus motifs across species. The individual isogene families showed additional consensus motifs, indicating that the selection pressure holds individual amino acids constant in these repeats. This may be a hint of the receptor function of these antigens rather than a presentation of random epitopes, generating the variability of these surface molecules.

## 1. Introduction

Variable surface antigen coats are a key feature of parasitic and free-living unicellular organisms. Due to their different life strategies, antigenic systems as well as their regulation mechanisms differ among different species but also show several common strategies [1]. The major mechanism for antigenic variation is the presentation of variable epitopes to the environment: this can be seen as molecular camouflage, e.g., the antibodies produced against these surface proteins can no longer opsonize the pathogen. A prominent example of this strategy is trypanosomes, which evolved an additional mechanism to enhance antigenic variability. Since most antigenic systems rely on multigene families whose members show mutually exclusive expression, *Trypanosoma brucei*, for instance, increases its variability by permanent segmentational gene conversions to create new surface antigen genes [2].

In *Plasmodium*, another strategy and mode of action of surface antigens can be observed. The phenomenon of sequestration is believed to be a key factor in the virulence of *P. falciparum* since other *Plasmodium* species do not show this behavior [3]. Sequestration means the adhesion of infected erythrocytes to a variety of host cell types, e.g., to endothelial cells, thus enabling parasite growth in hypoxic postcapillary vessels and inhibiting erythrocyte demolishing in the spleen [3]. The erythrocyte membrane protein (PfEMP1) responsible for sequestration has antigenic properties and the humoral immune response of infected individuals can target infected erythrocytes for destruction; the parasite evades this by switching the PfEMP1 variant encoded by the var-multigene family [4]. Research on different types of var genes’ PfEMP1 proteins demonstrated that some of them have different binding affinities to tissue-specific determinants, thus determining the tissues in the body that serve as a depot for infected erythrocytes [5,6,7]. Thus, the PfEMP1 proteins hold the capacity for both the specific binding of receptors and the presentation of variable epitopes for molecular hiding.

In addition, the free-living ciliate *Paramecium* undergoes programmed antigenic variation [8]. Surface antigen (SAg) expression has been studied in this model organism for a long time; however, most knowledge is limited to the laboratory strains, namely stock 51 of *Paramecium tetraurelia* and strains 156 and 168 of *Paramecium primaurelia*. The expression and inheritance of serotypes have been subject to research for many decades [9] because the mechanism of the mutually exclusive expression of the SAg multigene family, as well as the inheritance of the serotype to sexual progeny, remains hardly understood. Components of the RNAi pathway appear to be involved in serotype expression [10] and indeed SAg genes produce lots of small RNAs but their precise function again remains elusive [11].

*Paramecium* surface antigens are high-molecular GPI-(glycosylphosphatidylinositol)-anchored proteins [12] with a characteristic cysteine periodicity throughout the entire protein [13], which has also been reported for other organisms. In all SAgs, N- and C-terminal areas seem conserved and alignments of different proteins show less conservation in the central part of the proteins, which in some SAgs, consists of internal tandem repeats [14]. Given the fact that the internal regions (i) show a high dN/dS ratio even when SAgs are encoded by different alleles and (ii) are exposed to the medium, whereas the conservative marginal regions are hidden in the tertiary structure, this led to the conclusion that the immunologically relevant epitopes are located in the central region [15,16].

An unsolved question is the function of antigenic variation in free-living protists, especially free-living ciliates [17]. An involvement in predator–prey recognition was suggested [18] and this would indeed be a suitable explanation, but we do not know anything about this occurring in wildlife. Moreover, nothing is known about serotype expression in nature and all studies were carried out only with laboratory cultures, to the best of our knowledge.

SAgs in *Paramecium* might also be receptors. All cysteine-rich proteins on the cell surface have so far been reported to be GPI-anchored, which allows for cleavage by endogenous phospholipase C, thus shedding the SAg coat into the medium [19,20,21]. The anchor might also be involved in signal transduction. For instance, the addition of homologous antibodies to living paramecia induces serotype switches [22] and this was also reported recently for several other organisms, e.g., *Giardia*, *Trypanosoma*, and the ciliate *Tetrahymena* [23]. Furthermore, the GPI-anchored folate receptor on the *Paramecium* surface was shown to confer a folate-specific chemoresponse [24]; thus, GPI-mediated signal transduction appears to exist in *Paramecium*.

As mentioned above, only a few genes have been annotated to describe serotypes in *Paramecium prim-* and *tetraurelia*. Although their genomes evolved from three successive genome duplications, with approx. 68 percent of the genes remaining in the duplicates (ohnologs) [25], the SAg genes appear not to have evolved from ohnologs [26]. The SAg family in *P. tetraurelia* was previously shown to consist of isogene families, e.g., the 51D gene family [27], which also exists in *P. primaurelia* [28]. The nomenclature of serotypes involves the strain number in association with letters for the individual SAg genes. Only 11 genes for expressed SAgs are known, three in *P. primaurelia* 156G/168G [14,16], 156D [28], and 156S [29], and eight in *P. tetraurelia* (without non-expressed isogene families) 51A [30], 51B [31], 51C [13], 51D [27], 51G [32], 51H [33], 51I, and 51J [26]. Some of these SAgs have been described as consisting of internal tandem repeats building the immunologically relevant epitopes [16]; however, these repeats are not conserved [13]. An earlier screen of the *P. tetraurelia* macronuclear genome identified many more putative SAg genes, which remain unannotated as they have so far not been found to be expressed [26]. The lack of data for other *Paramecium* species is evident. The aim of this study was to screen the available genomes of several *Paramecium* species for SAg genes and figure out whether their SAgs have similarities and whether gene duplication events for certain isogene families also occurred in other genomes.

## 2. Materials and Methods

### 2.1. Identification of the SAg Dataset

**Figure 1:**
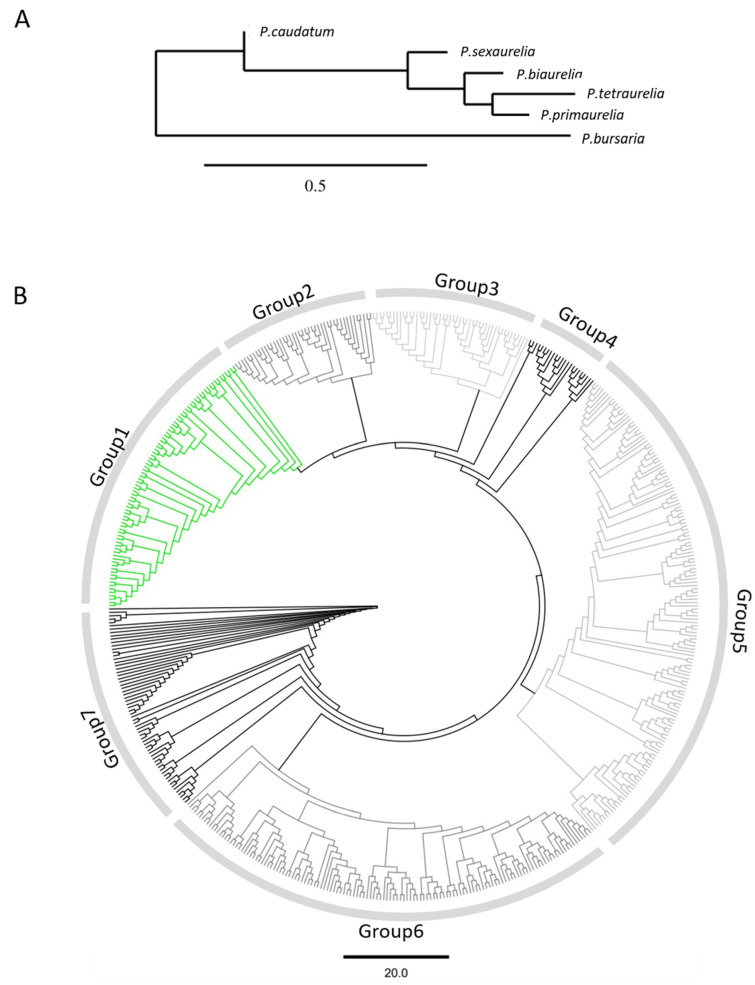
(**A**) Scheme of the evolutionary distance between the studied *Paramecium* species inferred from the mitochondrial COI gene sequence. The scale bar corresponds to 0.5 substitutions per site. (**B**) Cladogram of the neighbor-joining tree of the 548 identified putative SAgs. Scale bar represents substitutions per site.

Proteins of 6 *Paramecium* species were included in the analysis. Based on a set of 56 sequences of *P. tetraurelia* 51’s strain proteins that have been described as putative surface antigens in [26], we built a hidden Markov model with HMMER (http://eddylab.org/software/hmmer, accessed on 12 January 2021). Using this model, we screened the available MAC genome assemblies with *Paramecium*DB (all versions v1.0, except for *P. primaurelia* v.1.1) and proceeded only with proteins larger than 1000 amino acids. All proteins described here are shown with their accession numbers from *Paramecium*DB [34]. Accession numbers of proteins involve information about the species and strains: PPRIM.AZ9-3-*P. primaurelia strain AZ9-3*; PBIGN-*P. biaurelia strain V1-4*; PTET.51-*P. tetraulelia strain 51*; PSEX.AZ8-4-*P. sexaurelia strain AZ8-4*; PCAU.43c3d-*P. caudatum strain 43c3d*; PBUR.STL3-*P. bursaria strain STL3*. Multifasta files of the groups displayed in Figure 1B are available in the Supplementary Materials for this article.

### 2.2. Alignments and Phylogenetic Analyses

The 767 bp-long COI gene sequences retrieved from the mitochondrial genomes of the strains AZ9-3 (*P. primaurelia*), V1-4 (*P. biaurelia*), 51 (*P. tetraurelia*), AZ8-4 (*P. sexaurelia*), and 43c3d (*P. caudatum*) in ParameciumDB [34] were used to constrain the molecular phylogenetic scheme, demonstrating an evolutionary distance between the analyzed *Paramecium* species (Figure 1A). For *P. bursaria*, the COI gene sequence of strain K11-4B (GenBank MT078144), which is the same syngen as the strain STL3, was used. The phylogenetic tree was produced with the help of Phylogeny.fr [35]. The tree was computed using the bootstrapping procedure (500 bootstraps) and approximate likelihood ratio test method PhyML 3.1/3.0 aLRT. Maximum likelihood analysis was performed using the HKY model. All other analyses were carried out in Geneious Prime 2022.22.1. Alignments used Geneious Alignment mode (global alignment with free and gaps). Neighbor-joining trees used Jukes–Cantor genetic distance models with bootstrap re-sampling and 100 pseudoreplicates. For analysis of the scaffold synteny between *P. tetraurelia* and *P. primaurelia*, scaffolds that harbored either the D- or B-isogenes of both species were extracted and aligned with the Mauve Genome Aligner [36] plugin (Version 1.1.3) for Geneious Prime 2022.22.1, using the progressive Mauve algorithm with the default settings to identify the colinearity of the different parts of the scaffolds. To improve the overview, the positions of the B- and D-isogenes within the different scaffolds were added to the Mauve aligner output.

### 2.3. Prediction of Protein Characteristics and Motifs

Transmembrane domains were predicted using TMHMM-2.0 [37], and C-terminal GPI-anchoring signals were predicted using GPI-SOM [38]. We used the MEME suite version 5.4.1. [39] involving MEME for the discovery of recurring ungapped motifs [40] and SEA for further analysis of the identified motifs [41]. For MEME, the individual settings involved (i) any number of repetitions (anr) (ii) identifying 3 motifs, a 0-order model of sequences, and a range of 40–90 amino acid lengths of the motifs. Identified motifs were loaded into SEA (default settings) for analysis of individual groups.

## 3. Results

### 3.1. Identification of Putative SAgs in Different *Paramecium* Species

To screen the different *Paramecium* genomes for putative SAgs, we took advantage of the recent publication of macronuclear (MAC) genomes from several *P. aurelia* species and the *P. caudatum* and *P. bursaria* species [34]. We selected the species and omitted screening all available genomes because we aimed to first gain insight into a limited number of SAgs using some more-or-less related species inside the *P. aurelia* complex and two species outside the complex. *P. tetraurelia* is a model species for molecular genetics, whereas stock 51 is the most studied strain. Its MAC genome was published in 2006 and at that time, it was the first *Paramecium* genome resource [25].

In the case of *P. primaurelia*, the situation is complicated. Several SAg genes were published before from strains 156 and 168 originating from the USA. Unfortunately, these strains were not chosen for genome sequencing but instead, *P. primaurelia* strain AZ9-3, which was collected from Astrakhan Nature Reserve (Russia) in 2002 [42], was used. In earlier studies, this strain was used as a model for molecular karyotyping [43] and its MAC molecular karyotype was identical to that of strain 156 [44]. Later, we attempt to relate the known *P. primaurelia* sequence data to this strain. In addition, we chose *P. biaurelia* strain V1-4; *P. sexaurelia* strain AZ8-4, a more divergent species of the *P. aurelia* complex; *P. caudatum* strain 43c3d as a close relative of the *P. aurelia* complex, which, however, had not undergone the latest WGD; and finally, *P. bursaria* strain STL3, one of the early diverged *Paramecium* species representing an outgroup to the *P. aurelia*/*caudatum* cluster (Figure 1A).

We identified 548 putative SAgs in the 6 species and we approached an initial classification of them by an unrooted neighbor-joining tree based on amino acid sequences to visualize their similarities without a deeper insight into the evolutionary relationship. Figure 1B shows the resulting cladogram in which seven different groups can be distinguished. Some of them represent clear clusters separate from others and show shorter branches between individual members. Other groups need to be dissected at a later point. In the following sections, we aim to classify these groups by certain characteristics; however, we anticipated the green cluster in Figure 1B consisting solely of *P. bursaria* SAgs (see below).

### 3.2. Internal Tandem Repeats Are Not Present in All SAgs

**Figure 2:**
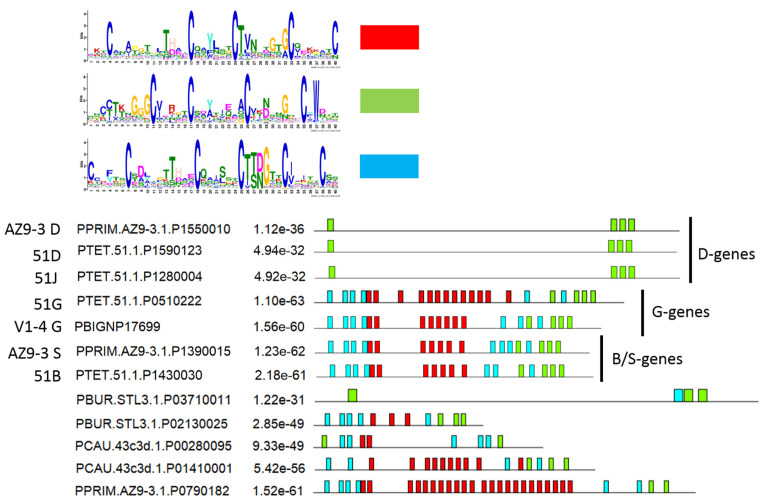
**Identification of recurring motifs in SAgs.** Three motifs were identified, each with more than 1000 sites in the entire dataset and with E-values of $4\times 10^{-1909}$ (red), $1.8\times 10^{-1868}$ (green), and $1.6\times 10^{-1299}$ (blue). The location of the motif sites is shown as exemplary for some SAgs involving D-, G-, B-, and S-SAgs from different species and also some unknown SAgs inferred from the genome data. Each block shows the position of a motif; the height of a block indicates significance—taller blocks are more significant, being proportional to the negative log of the positional *p*-value of each motif but truncated for a *p*-value of $1\times 10^{-10}$. All motifs have *p*-values below that threshold. Between the accession numbers and the scheme, the combined *p*-value match for the entire SAg is given.

Before continuing with the comparison of the seven groups, we analyzed one criterion that required additional characterization. Internal tandem repeats have been frequently described in some SAgs (51A, 51B, 51G, 156G, 168G, 156S) [14,16,29,30,31,45] but not in others (51D, 156D, 51C) [13,27,28]. A bioinformatics prediction of these repeats is difficult as they sometimes do not match perfectly and vary in proteins. We used a different approach and analyzed the entire dataset for recurring motifs using MEME [40]. By analyzing the complete dataset, three motifs with high significance were identified (Figure 2). All three revealed constant cysteine periodicity, which *Paramecium* SAgs are known for. The distribution of the individual motifs inside the polypeptide was shown to be exemplary for the individual SAgs of the different species (Figure 2). Although the blue and green motifs seemed to be concentrated in the marginal regions, the red motif showed a repetitive pattern inside the SAgs. This red motif indeed seemed to represent the internal tandem repeats. It was surprising to find a consensus motif of CTVNXXGTGC in the internal repeats across species and different SAgs because the internal regions have been described as being highly variable even in the comparison of SAgs in a single species [26]. However, the motif seemed to be reliably detectable in repeats containing SAgs. Figure 2 also shows the pattern of repeats for some previously described SAgs, which are the D genes including the 51J gene, which is believed to be a 51D gene duplicate [26]. The D genes have been described as being devoid of internal repeats contrary to, e.g., the G and B families of *P. primaurelia* and *P. tetraurelia*, which have been described as containing several internal repeats, as also seen in Figure 2.

### 3.3. An Attempt at the Categorization of SAg Groups

As our data now indicated that the presence of internal repeats could be another criterion for dissecting the individual classes of SAgs, we collected data on several parameters from the individual groups identified in Figure 1B. This included the length distribution, membrane anchoring, species composition, and presence of the internal consensus motif.

**Figure 3:**
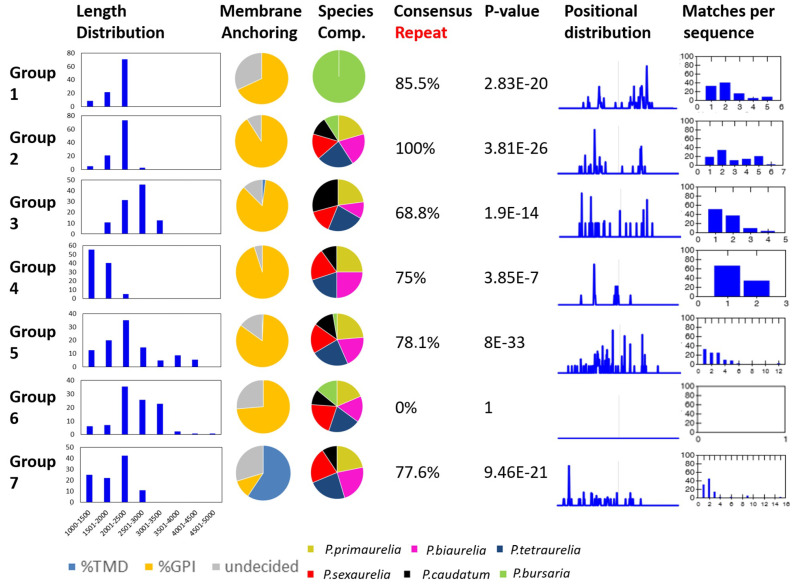
**Characteristics of the individual SAg groups.** The first column shows the length distribution of SAgs in amino acids. The bins are indicated at the bottom; the Y-axes show the percentages of SAgs that fall into one bin. The pie charts indicate the composition of each group in terms of species composition and membrane anchoring. The decision on transmembrane anchoring was the prediction of at least two transmembrane domains. GPI-anchoring prediction relies on the prediction of the C-terminal GPI-anchoring signal. The last columns refer to the analysis of the internal repeat motif (red motif) in the individual groups. SEA was used to analyze the enrichment of this motif in a dataset. The column “Consensus Repeat” indicates the percentage of primary sequences matching the motif. The *p*-value shows the optimal enrichment *p*-value of the motif but it is not adjusted for the number of motifs. The positional distribution of the motifs in the linear polypeptide sequence is shown in the respective column and the last column shows the number of matches per sequence as a percentage of the total sequences of a group.

Classically described serotype proteins are of high molecular weight. The average length of the coding region for previously known SAgs is 6.6 kb so the proteins have an average of 2.200 amino acids length. This fit very well for all groups (Figure 3), except for group 4, which was composed of only a few yet diverse proteins, and group 7, which included the transmembrane proteins (see below) and showed a broader size range.

Column two in Figure 3 summarizes the data on membrane anchoring predictions in terms of transmembrane domains and GPI-anchoring signals. In the undecided group, the algorithms were unable to predict either GPI- or transmembrane anchoring. Proteins with transmembrane domains were almost exclusively found in group 7. Classical SAgs, as well as many smaller cysteine-rich surface antigens, have been shown to be GPI-anchored [12,15,20,24] and this fit to all other groups. Transmembrane domain-containing proteins with cysteine repeat structures have been reported in *P. tetraurelia* before, but through just a genomic screen, nothing is known about their expression, localization, and function [26]. Our data suggest that these proteins exist in all *Paramecium* species, except for *P. bursaria*. Maybe they are indeed missing in this species or they could be too divergent to be identified in our screen.

The third column in Figure 3 shows the species compositions of the groups, indicating that group 1 contained SAgs of *P. bursaria* only. This species indeed had a different distribution in the groups because *P. bursaria* SAgs were identified in groups 2, 5, and 6 but not in groups 3, 4, and 7. SAgs of all other species including *P. caudatum* were identified in all groups, except for group 1.

We used SEA [41] to screen all genes for the presence of the motif associated with the internal repeats (labeled in red in Figure 2). Figure 3 shows these results in the last four columns for the individual groups. The motif was identified in the proteins of all clusters, except for group 6. The highest significance for enrichment was apparent for group 5, which is the group containing the B, G, and S serotypes, which were described as containing internal repeats. The repeats were accumulated in the central regions. Group 3 (including the D and H genes) and group 4 had a high percentage of proteins with predictions of internal tandem repeats but with much lower significance; also, the repeat frequency suggested one or two matches per protein only. In groups 1, 2, and 5, the repeat frequency showed a higher percentage of proteins with a higher number of matches per protein, thus meaning more repeats per protein than in groups 3 and 4.

The problem with interpreting these data is that nothing is known about the functional relevance of the internal repeats. However, our data indicate that the presence and number of internal repeats at least help to describe the differences between the groups of different SAgs. *P. bursaria* seems interesting in several aspects. Group 1 proteins show all the characteristics of classical SAgs, that is, GPI-anchoring, size, and internal repeats. The distribution of *P. bursaria* SAgs among the clusters suggests the evolution of some specific or divergent SAgs. However, it is interesting that *P. bursaria*-specific SAgs also had good matches with the consensus motif and possessed highly repetitive structures.

### 3.4. Group 3 Contains the D and H SAg Isogene Families

**Figure 4:**
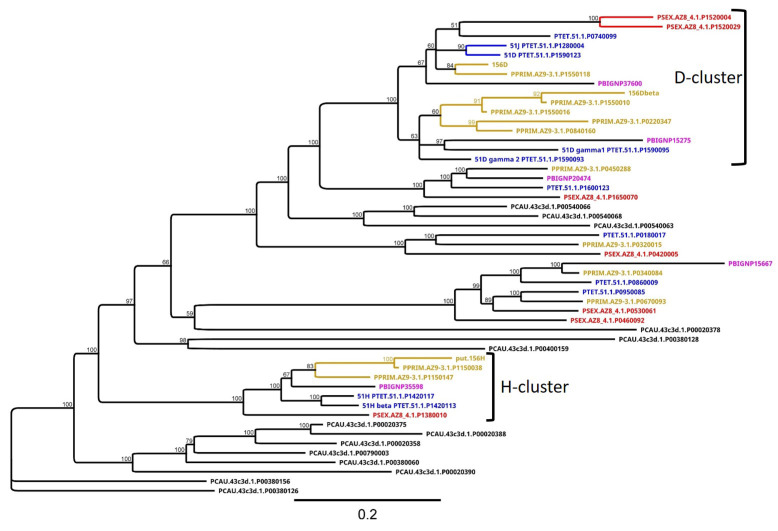
**Neighbor-joining tree of cluster 3, which contains the D and H surface antigens.** The known genes for *P. primaurelia* (156D, 156D beta, the putative 156H) are spiked into the alignment to identify the orthologs in the *P. primaurelia* AZ9-3 strain. The identified proteins that correspond to known SAgs in *P. tetraurelia* are indicated (51D, 51J, 51D gamma1, 51D gamma2, 51H alpha, and 51H beta). *P. primaurelia* is colored in yellow, *P. biaurelia* in pink, *P. tetraurelia* in blue, *P. sexaurelia* in red, and *P. caudatum* in black. Scale bar represents substitutions per site; branch label indicates percentage consensus support of 100 pseudoreplicates.

We focused on the groups that contained SAgs that had been described earlier to relate our data to the associated molecular data. Figure 4 shows a tree of group 3 with two rather discrete clusters containing the D and H isogene families. We need to mention that we spiked in known SAg sequences from *P. primaurelia*. As mentioned above, most molecular work was conducted with *P. primaurelia* strains 168 and 156. To connect the proteins of *P. primaurelia* strain AZ9-3 to the known sequences, we included the 156D isogenes and SAg 156H in the alignments.

Looking at the D cluster, one can identify all *P. aurelia* species. Thus, D genes seem to be *P. aurelia*-specific. It was described earlier that in most instances, one gene of an isogene family is expressed. These genes are called the alpha genes, e.g., 51D alpha [27]. Further members are classified by subsequent Greek letters. The alpha genes, that is, the expressed SAgs 51D, the AZ9-3 ortholog to 156D, and SAg 51J, which is an independent serotype with high similarity to 51D (maybe a former duplicate), create one cluster that is separated from the non-expressed isogenes; the separation of the expressed genes from the non-expressed isogenes can be seen later for other SAgs (see below). Our screen identified the known *P. tetraurelia* isogenes 51D gamma 1 and 2 and many more SAgs with high similarity to the fragment of the 156D beta isogene, suggesting that the expansion of these D isogenes in *P. primaurelia* resulted in many more gene copies compared to all other species. For the H cluster, we observed two H genes in *P. tetraurelia* and *P. primaurelia*, respectively. *P. sexaurelia* and *P. biaurelia* revealed only a single H ortholog.

This indicates that SAg isogene families have different duplication levels in different species within the *P. aurelia* complex.

### 3.5. Diversification of Internal Repeats in SAg Subfamilies

**Figure 5:**
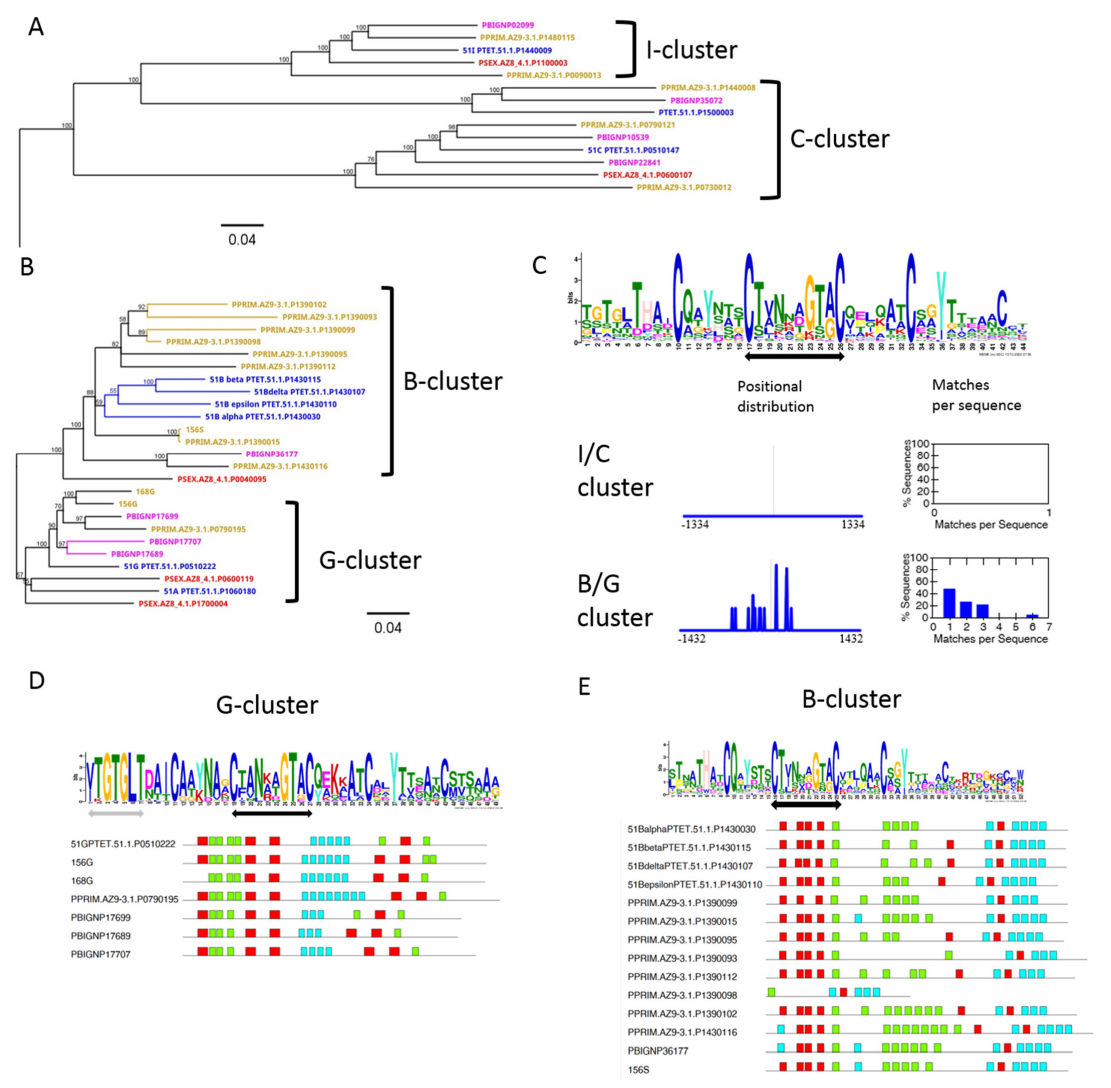
**A deeper analysis of cluster 5 containing the I, C, B, and G SAgs**. (**A**,**B**) show subtrees of the cluster containing *P. tetraurelia* 51C and 51I SAgs (**A**) and the *P. tetraurelia* 51A, 51G, 51B family, and *P. primaurelia* G and S SAgs (**B**). (**C**) shows the motif of internal repeats for the genes in the subclusters in (**A**,**B**), and the positional distributions and motif frequencies in the I/C cluster and the B/G cluster. (**D**,**E**) show the motif of the internal repeats dissecting the G (**D**) from the B cluster (**E**). The motif is shown for the internal repeats only, which are indicated in blue for the G cluster and in green for the B cluster. Please note that the colors are used differently for the motif blocks in (**D**,**E**). Below the motif, the distribution is shown for the individual SAgs. In all three motifs in this figure, the common CTXNXXGTAC motif is indicated by the black arrows. The G-cluster-specific YTGTGLT motif is highlighted by the grey arrow.

**Figure A1:**
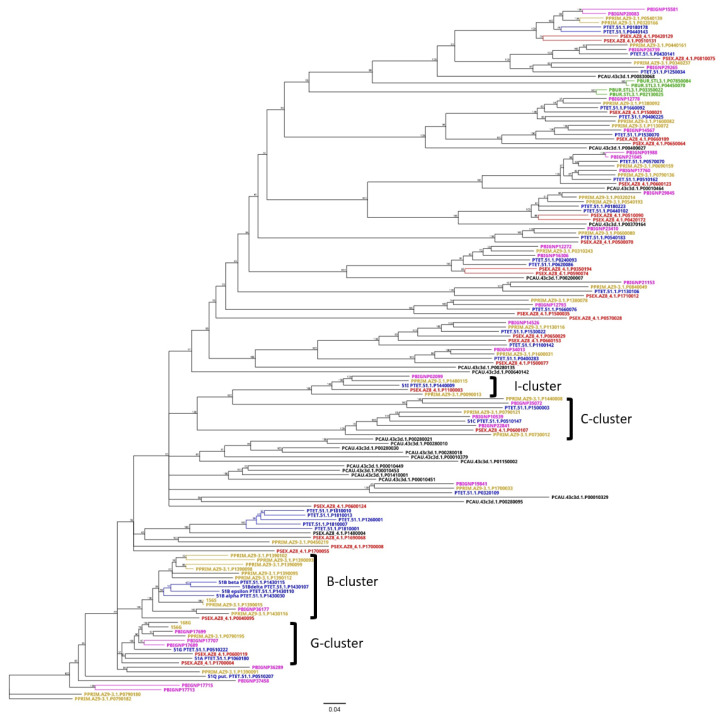
Neighbor-joining tree of group 5 with the SAg 51B, the B isogene family, and SAgs 51G, 51I, 51C, and 51A. The known genes for *P. primaurelia* (156S, 168G, and 156G) are spiked into the alignment to identify the orthologs in *P. primaurelia* strain AZ9-3. Identified proteins that correspond to known SAgs in *P. tetraurelia* are indicated (51B isogene family, 51A, and 51G). *P. primaurelia* is colored in yellow, *P. biaurelia* in pink; *P. tetraurelia* in blue, *P. sexaurelia* in red, and *P. caudatum* in black.

Group 5 (Figure 1B) was composed of SAgs with clear separation from the other groups. This cluster contained many of the previously described SAgs (Figure A1). Figure 5A shows the subtree of the *P. tetraurelia* 51I and 51C SAgs. Both had relatively close orthologs in other *P. aurelia* species but there seemed to be no intraspecific duplicates. This was different for the B and G clusters in Figure 5B, where many different isogenes can be identified. Compared to the I and C clusters, the branches were much shorter, indicating higher similarity among SAgs and therefore a more recent duplication origin.

B genes showed many duplicates in *P. primaurelia* and *P. tetraurelia*. We identified one more close ortholog only in *P. biaurelia* and this seemed to be a single copy gene. Again, we spiked known *P. primaurelia* SAgs into the alignments and we identified an AZ9-1 ortholog to the 156S allele.

A B allele has not been reported before for *P. primaurelia*. Together with serotypes D and G, the S serotype was instead described as being present in almost all studied *P. primaurelia* species [46], and serotype S (alleles 156S and 60S) was further described as cross-reacting with 51B antiserum (G. Beale personal communication). The immunological cross-reaction was later confirmed by sequence comparisons [29]. We therefore hypothesize that 156S (and consequently, AZ9-3S) is the ortholog to 51B. In addition, we could conclude further that AZ9-3S (P1390015) is the alpha gene of the AZ9-3 S isogene family, being separate from isogenes. The separation of alpha SAgs to isogene duplications can also be observed for the D genes in Figure 4; however, this is not supported by the branch support values for *P. tetraurelia* B genes (Figure 5B). It might be tempting to speculate that selection pressure acts on the expressed alpha isogenes, whereas lowly expressed or silent isogenes could diverge rapidly.

In contrast to the high duplication level of B genes, this is not the case for G genes. Our dataset indicated one allele in *P. primaurelia* AZ9-3 and one allele in *P. tetraurelia*(51G) but three genes in *P. biaurelia*. The cluster also contained the 51A allele, which did not show close relatives in any species. In summary, we saw large isogene families with varying numbers of members in different species. The D, B, and H isogenes showed similar but not identical numbers of members in *P. primaurelia* and *P. tetraurelia*, which are the closest relatives in our comparison. This suggests that these two species tend to duplicate the same genes. The G genes were amplified only in *P. biaurelia*, and this species showed only a single D duplicate rather than an isogene family. This confirms that the species indeed showed different duplication levels of individual SAgs.

The presence of the I and C SAgs in cluster 5 together with the B, S, and G SAgs revealed an additional aspect to consider. It was previously described by Nielsen et al. that the *P. tetraurelia* C SAg does not have any internal repeats, whereas 51B does [13]. This means that our identified groups in Figure 1B require further dissection for internal repeats.

We used the subset of SAgs from Figure 5A,B to define a more specific consensus motif for the internal repeats (Figure 5C) and used SEA to show its enrichment in the I/C and the B/G clusters. Figure 5C shows that the motif could not be detected in the I/C cluster. We also did not identify any other class of repeats in the I/C cluster with MEME. Thus, we conclude that not all of group 5 showed internal repeats but they occurred only in individual subclusters.

We further aimed to see whether the different subclusters showed differences in the repeats and used MEME for a new motif prediction from the G and the B clusters separately. Figure 5D,E show that these submotifs indeed contain the CTVNXXGTXC consensus (arrow) and also show additional amino acid preferences, e.g., a clear YTGTGLT motif in the G cluster, which could not be detected in the B cluster. The B cluster motif consisted of individual highly conserved amino acids, which were not conserved in the G cluster. These sequence analyses suggested that internal tandem repeats occurred in different subclasses of SAgs but not in all of them. If internal repeats were detected in a sublcuster, all SAgs from different species contained these repeats. Thus, the distinct serotype proteins showed different motifs in the internal repeats and these motifs were conserved across different species.

### 3.6. Chromosome Synteny: 156S Is the Ortholog of 51B

**Figure 6:**
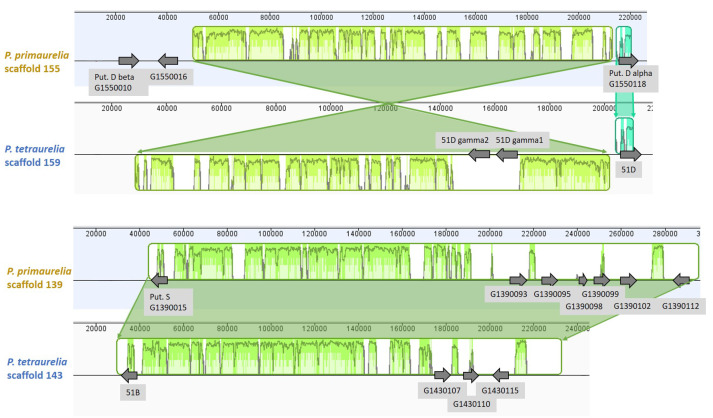
**Synteny between scaffolds of*****P. primaurelia*****and*****P. tetraurelia*****harboring D isogenes (upper part) and B isogenes (lower part)**. The scaffolds are visualized by the individual rulers giving the length position in bp. The similarities among the scaffolds are shown by alignment scores within the highlighted boxes (green/teal). The positions of the identified SAg genes are shown by grey arrows. Putative orthologs are included according to the clustering of the SAgs of strain 156 in the neighbor-joining tree. Scale bar represents substitutions per site; branch label indicates the percentage consensus support of 100 pseudoreplicates.

The *Paramecium* genome has undergone several rounds of whole genome duplication. *P. aurelia* has undergone three individual rounds [25]. As a result, many genes still exist as duplicates. The initial analysis of SAg genes in the *P. tetraurelia* MAC revealed only a few ohnologs but many single-copy genes. All of the classically described serotype genes were located in subtelomeric regions [26]. Gene duplicates, as discussed for the D, H, or B isogene families, were located on the same MAC chromosomes arising from duplication events other than the WGD. To see whether the isogenes of a SAg family arose before the last WGD, we analyzed the synteny of orthologous chromosomes as shown in Figure 6. The upper comparison shows the two D-gene-containing chromosomes between *P. primaurelia* and *P. tetraurelia*. The respective alpha gene is located in the right subtelomeric region. The chromosome shows an inversion and different positions of the two duplicates. We also performed a comparison of the two chromosomes containing the *P. tetraurelia* B and the *P. primaurelia* S SAgs, respectively. Both chromosomes showed a high degree of similarity and synteny, indicating orthologous chromosomes. The putative AZ9-3 S allele showed the same subtelomeric localization as the 51B allele, which led us to conclude that the *P. primaurelia* S serotype gene is orthologous to the *P. tetraurelia* B serotype gene. In both chromosomes, we observed internal duplicates that disrupted the conserved synteny between the two chromosomes. Both examples suggest that the gene duplication events leading to the serotype isogenes families are younger than the last whole genome duplication. This would mean that both species tend to amplify these genes independently.

## 4. Discussion

We need to start with the limitations of this approach. One quite evident aspect is that we could not analyze most of the groups deeper. The reader can see that we focused on groups 3 and 5 as they contained genes for which we have some literature background. Then, we discussed group 1, which was formed by the *P. bursaria*-specific SAgs. In contrast to *P. caudatum*, most of the *P. bursaria* SAgs clustered in group 1. As these share the characteristics of the SAgs described from the *P. aurelia* species, it was reported recently that the SAgs belong to the six gene families with the highest duplication levels in the *P. bursaria* MAC [47]. We concluded that this species evolved some divergent SAgs, maybe due to the totally different autecology. *P. bursaria* hosts symbiotic algae and is a mixotrophic ciliate, whereas *P. aurelia* feeds on bacteria. As a result, they have different habitats; *P. bursaria* occurs throughout the water column, whereas *P. aurelia* preferentially resides closer to the substrates suitable for bacterial growth. In addition, *P. bursaria* is thought to have a higher genetic diversity because of the absence of autogamy (which leads to homozygous individuals) and the multipolar mating system [48]. Thus, the genetic and ecological aspects of both are quite different, which may account for the different needs of the serotype systems.

In addition to these three groups, the discussion of groups 2, 4, 6, and 7 appeared difficult, as we could not relate these proteins to any previous data. As mentioned, for group 7, which included transmembrane proteins, we did not have either molecular data on their subcellular localization or any molecular data at all.

The second limitation of our study is that we relied on gene models. This means that we might have missed, e.g., shortened genes due to incorrect ORF annotations. Indeed, we observed some SAgs that were likely too short, for instance, those of the B isogenes in Figure 5E, but given both the length distribution of the SAgs in the clusters, as well as the fact that we found a high percentage of C-terminal GPI signals, there seemed to be only a few artificially shortened genes in our dataset. The third limitation is that we searched for the cysteine periodicity described for the *P. aurelia* SAgs. There could be SAgs with other characteristics. However, we identified a large number of putative SAgs in these species so there at least seems to be a serotype system that is comparable.

We could have also missed SAg genes due to a combination of sequencing depth and heterogeneity of MAC chromosome ends. The MAC genome is newly developed from two haploid MIC genomes during a sexual process and this process always causes certain heterogeneity of the MAC chromosomes, as reviewed in [49]. One aspect of heterogeneity concerns the MAC chromosome ends; these may exist in different lengths, and the published scaffolds only represent the longest possible scaffolds, ignoring the shorter versions [50]. The recent deep sequencing of different MAC genomes revealed many MAC-variable regions at chromosome ends [51] and this heterogeneity influenced our study here in several aspects. First, we may have missed SAgs that were located in MAC-variable regions and not present in the genome assembly. There are likely more SAgs in the MIC that did not make it into the MAC. For this assumption, there was some evidence in *P. primaurelia*: a G pseudogene was identified before the genome was published in an under-amplified region [52]. The authors identified the low-copy chromosome, which could be an alternative chromosome end containing the G pseudogene. Interestingly, they also found a gene upstream of this G pseudogene, with homology to an upstream gene of the G gene. The synteny between both regions may suggest that these are ohnologous chromosomes; the WGD duplicate of the G gene would be still present in the MIC genome, but the MAC chromosomal end of the paralog would not be fully amplified in the MAC, only that of the intact G SAg. This may be the reason why we do not see the ohnologs of most of the SAgs in MAC genomes. Before getting lost in speculation here, one needs to screen the MIC genomes, which are not available yet, for the SAg genes to understand their evolution. The example of the under-amplified G pseudogene and its synteny to the expressed G gene in *P. primaurelia* suggests that SAg ohnologs still reside in the MIC, whereas their absence in the MAC may be controlled by epigenetic mechanisms controlling MAC chromosome heterogeneity and the amplification of MAC chromosomes and their parts. Similarly, the *P. tetraurelia* 51A gene in the MAC is located close to the telomere and its amplification; in other words, the MAC chromosome version, which includes the 51A gene instead of a shorter version, can be epigenetically controlled by the presence/or absence of the 51A gene in the parental MAC [53,54]. The lack of SAg ohnologs in the MAC could therefore be due to an epigenetic effect controlling their presence in the MAC and they could still be present in the MIC. The previous screen of *P. tetraurelia* SAgs revealed what was known for single genes before, e.g., that the expressed SAgs are located in the subtelomeric regions but not the isogenes [26]. We cannot faithfully expand this analysis to the other species analyzed here, but the question remains, that is, whether this genomic localization is due to their evolution or due to their expression mechanism. Subtelomeric recombination could be an argument for SAg evolution. However, meiosis occurs with MIC chromosomes, which can account for the subtelomeric SAgs on MIC chromosomes only. We need to consider that MAC chromosomes can be dynamic, even the SAgs in the centers of the acentromeric chromosomes can be subtelomeric in a subfraction of the MAC chromosomes, e.g., as shown for SAg 51C [55]. An involvement of the subtelomeric localization in the regulation of serotype expression, meaning the parallel activation of a single gene while all other SAgs remain silent, appears likely, as a knockdown of RNAi components revealed the co-activation of MAC subtelomeric SAgs only [26].

We still do not know the origin of the intrachromosomal duplicates. However, our data shows that duplicates are not ohnologs and they likely evolved more recently and after the last WGD. Thus, the SAg family appears to have evolved quite fast. As suggested previously [26], unexpressed isogenes may diverge rapidly from their origin and may become activated as new SAgs when MAC heterogeneity allows them a subtelomeric position by activation of a new telomere addition region. However, this does not explain the duplication mechanism or the reason why we see most isogenes on the same MAC chromosome.

Our data indicate two additional features: alpha genes of isogene families seem to be separated from non-expressed isogene duplicates and we see some species-specific isogene copies, e.g., the G gene copies in *P. biaurelia*. Some SAgs seem to have different duplication levels in different species. The similarities between *P. primaurelia* and *P. tetraurelia* are evident, for example, the same SAgs have isogenes (D, H, B) and the same SAgs are single-copy genes (I, C, G). These similarities may reflect the close relationship between both species. Still, the synteny of orthologous chromosomes among species suggests that they independently started to create gene copies of the isogene families, which may be due to a common selection pressure acting on both species.

Finally, we want to return to the function of SAgs as introduced in the Section 1. As mentioned in the introduction, antigenic variation may be a mechanism for the presentation of variable epitopes and/or the presentation of constant epitopes for ligand detection. As explained for PfEMP1, proteins can also fulfill both functions.

Our screen identified three interesting aspects: (i) internal repeats were not present in all SAgs, (ii) internal repeats showed a consensus motif across groups and species, and (iii) internal repeats showed sub-diversification in isogene families across species. We used a rather unconventional approach for repeat detection via motif recognition. This implied a degree of similarity among the repeats and the species. This is what we found with the CTVNXXGXGC motif. Given that the internal repeats were exposed to the outside and that valine was the only hydrophobic amino acid, this motif could be immunologically relevant or it could represent a ligand binding site. What we can decide here is that this motif was not relevant for immobilization or in vivo determination because then, all serotypes with internal repeats would cross-react, which is not the case. Otherwise, the motif could hold a structural function because all amino acids are relatively small, which makes it a bit reminiscent of linker peptides, which are usually used to relax structures in fusion proteins. Maybe the latter would indeed be suitable for SAg tertiary structures because they are believed to be quite densely packed. Their conserved N- and C-terminal areas are hidden, for instance, monoclonal antibodies against the C-terminal area require the rupture of intramolecular disulfide bonds to recognize the protein [56]. Assuming a high level of disulfide bridges in a protein with more than 10% cysteine could require lots of flexible linkers. This could also be true for the two other motifs in the marginal regions because their consensus is also mostly accumulated by glycine and threonine. It is interesting that we can find more consensus motifs in the internal repeats when we use smaller datasets of individual isogene families. The finding, e.g., of the YTGTGLT motif in the G cluster repeats, suggests that individual serotype proteins indeed specialize their exposed amino acids and that the central repeats consist of a general motif and an individual motif. If selection pressure maintains the individual motif in different species, this could be an argument for a receptor function and would make sense as these motifs are repeated. One could speculate here that SAgs with internal repeats are used for signal reception from the environment, whereas SAgs without internal repeats may be used for classical antigenic variation, meaning the molecular camouflage for displaying random epitopes to the environment. However, without any data on which serotypes are expressed in wildlife, this cannot be experimentally proved.

## Data Availability

All data were extracted from *Paramecium*DB (https://paramecium.i2bc.paris-saclay.fr/); the Supplementary files of this article contain gene lists with the accession numbers of *Paramecium*DB [34].

## Supplementary Materials

The following supporting information can be downloaded at https://www.mdpi.com/article/10.3390/microorganisms10122378/s1, group1.fas; group2.fas; group3.fas; group5.fas; group6.fas; and group7.fas.
